# Orientation-Dependent Mechanical Behaviors of BCC-Fe in Light of the Thermo-Kinetic Synergy of Plastic Deformation

**DOI:** 10.3390/ma17102395

**Published:** 2024-05-16

**Authors:** Yu Liu, Jinglian Du, Kunyu Zhang, Kangxu Gao, Haotian Xue, Xiao Fang, Kexing Song, Feng Liu

**Affiliations:** 1 State Key Laboratory of Solidification Processing, Northwestern Polytechnical University, Xi’an 710072, China; 2 Analytical & Testing Center, Northwestern Polytechnical University, Xi’an 710072, China; 3 Henan Academy of Sciences, Zhengzhou 450046, China

**Keywords:** BCC-Fe, anisotropic mechanical behaviors, thermo-kinetic synergy, dislocation motion, deformation mechanisms

## Abstract

The orientation-dependent mechanical behaviors of metallic alloys are governed by deformation mechanisms, but the underlying physics remain to be explored. In this work, the mechanical responses along different orientations and behind the mechanisms of BCC-Fe are investigated by performing molecular dynamic simulations. It is found that the mechanical properties of BCC-Fe exhibit apparent anisotropic characteristics. The <100>-oriented BCC-Fe presents a Young’s modulus of *E* = 147.56 GPa, a strength of *σ_y_* = 10.15 GPa, and a plastic strain of *ε_y_* = 0.084 at the yield point, whereas the <111> orientation presents *E* = 244.84 GPa, *σ_y_* = 27.57 GPa, and *ε_y_* = 0.21. Based on classical dislocation theory, the reasons for such orientation-dependent mechanical behaviors are analyzed from the perspective of thermo-kinetic synergy upon deformation. It turns out that the anisotropic mechanical responses of BCC-Fe are associated with the magnitude of the thermodynamic driving force (Δ*G*) and kinetic energy barrier (*Q*) for dislocation motion, which dominate the corresponding deformation mechanism. Compared with the low Δ*G* (6.395 GPa) and high *Q* (11.95 KJ/mol) of the <100>-oriented BCC-Fe dominated by deformation twinning, the <111> orientation governed by dislocation slip presents a high Δ*G* (17.37 GPa) and low *Q* (6.45 KJ/mol). Accordingly, the orientation-dependent deformation behaviors of BCC-Fe are derived from the thermo-kinetic synergy for dislocation motion.

## 1. Introduction

Body-Centered Cubic (BCC) metals, such as Fe, Mo, W, and Ta, are widely used in human lives and industrial production due to their excellent plasticity and ductility within a large deformation scope and wide temperature range [1,2,3]. Because of the abundant iron ore resources and mature smelting techniques, BCC-Fe and its alloys have become some of the most important metallic structural materials [2]. However, the low strength and hardness of BCC-Fe greatly restrict its industrial applications. Therefore, it is both scientifically important and technologically significant to improve the comprehensive mechanical performances of BCC-Fe and its alloys.

As two crucial mechanical indexes of metallic alloys, strength and ductility usually present exclusive behaviors [4], where strength enhancement inevitably is accompanied by a sacrifice in terms of ductility, and vice versa. How to break the original strength–ductility trade-off of BCC-Fe by clarifying the underlying physics becomes a critical point for the development of advanced Fe-based alloys with ideal strength–ductility combinations. In principle, the mechanical performances are reflected by the deformation mechanisms, which can be described by variations in the atomic structure during deformation [5,6,7,8]. For instance, Harding [9] shown that, under conditions of room temperature and low strain rates (10^−3^ s^−1^), the plastic deformation of bulk BCC-Fe occurs via dislocation slip. Both an increase in the strain rate and a decrease in deformation temperature can promote the formation of deformation twinning, the amount of which depends on the crystal orientation of BCC-Fe single crystals [9]. Wang et al. [10] observed the deformation twinning from experiments during plastic deformation of BCC-W nanowires at room temperature and low strain rate conditions. Healy et al. [11] reported the compression–tension asymmetry of BCC-Fe nanowires, where the plastic deformation proceeds by dislocation slip during compression, while the generation and motion of twin boundaries during tensile deformation. Sainath et al. [12,13] pointed out that the deformation behaviors of BCC-Fe single crystals depend on the monocrystal size and orientations. Both experimental and theoretical studies have shown that the crystal orientations have considerable influences on the deformation mechanisms of BCC crystals, where the competition and combination of different plastic deformation modes determine the orientation-dependent mechanical behaviors [9,10,11,12,13]. However, the potential mechanisms behind such orientation-dependent mechanical responses, along with the strength–ductility trade-off correlations of BCC-Fe, remain elusive.

In general, the plastic deformation process can be uniformly described as atomic kinetic behaviors triggered by a thermodynamic driving force [14,15,16,17]. Moreover, the thermodynamic and kinetic behaviors of metallic alloys during plastic deformation have mutually exclusive correlations similar to strength and ductility [15,16,17]. Thermodynamics and kinetics of dislocation behaviors dominate the plastic deformation mechanisms, and thus determine the mechanical performances of BCC-Fe [18]. In the present work, the mechanical properties and the plastic deformation mechanisms of BCC-Fe along different crystal orientations are investigated. The anisotropic mechanical behaviors are clarified from the perspective of thermo-kinetic synergy for dislocation motion. Firstly, the atomic structure and mechanical responses of BCC-Fe under different tensile conditions are obtained by performing molecular dynamics (MD) simulations. Then, the plastic deformation mechanisms of BCC-Fe along different orientations are clarified. Finally, the key thermo-kinetic parameters for dislocation motion of BCC-Fe under different tensile conditions are analyzed. The remaining parts of this work are organized as follows. In Section 2, the details of MD simulations of BCC-Fe are introduced. In Section 3, the results of the orientation-dependent mechanical responses and plastic deformation mechanisms of BCC-Fe under different tensile conditions are presented and analyzed. In Section 4, the thermodynamic driving force (ΔG) and kinetic energy barrier (*Q*) of dislocation motion are calculated, and the thermo-kinetic reasons for the anisotropic mechanical behaviors and strength–ductility trade-off relationship of BCC-Fe are discussed. Our investigations clarify the physical mechanisms behind the orientation-dependent mechanical behaviors of BCC-Fe, and provide a new perspective for understanding the strength–ductility trade-off phenomenon of metallic alloys.

## 2. Details of Structural Models and MD Simulations

**Figure 1:**
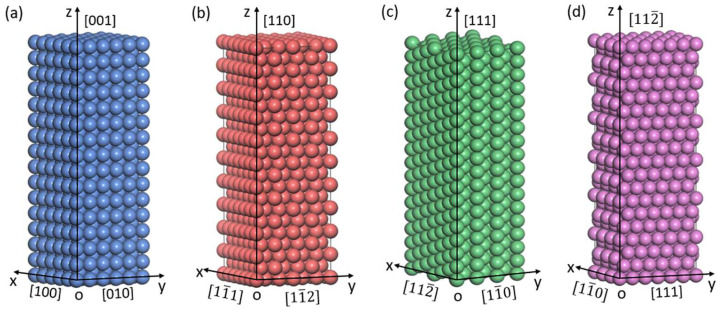
Schematic illustration for the atomic structural models of BCC-Fe along different tensile directions (z axis), including (**a**) <001>, (**b**) <110>, (**c**) <111>, and (**d**) <11$\bar{2}$> orientations.

The BCC-Fe supercells used in the MD simulations were 60 nm in the z direction and 20 nm in the x- and y directions (i.e., 70 a × 70 a × 210 a, with a = 2.855 Å being the lattice parameter of BCC-Fe [19]). Correspondingly, the simulation box contained ~2 million Fe atoms in the BCC crystal lattice. To investigate the mechanical behaviors and the according deformation mechanisms of BCC-Fe under different tensile conditions, three sets for MD simulations were used in the present work with reference to the simulation conditions commonly used in early research, including (i) the <100>-oriented BCC-Fe tensile deformation at temperature of *T* = 300 K and strain rates of $\dot{\varepsilon }$ = 1 × 10^8^ s^−1^, 1 × 10^9^ s^−1^, and 1 × 10^10^ s^−1^; (ii) the <100>-oriented BCC-Fe tensile deformation at $\dot{\varepsilon }$ = 1 × 10^8^ s^−1^ and *T* = 100 K, 300 K, and 600 K; and (iii) the tensile deformation along <100>, <112>, <110>, and <111> orientations of BCC-Fe at *T* = 300 K and $\dot{\varepsilon }$ = 1 × 10^8^ s^−1^. The corresponding coordination systems, the crystal orientation definitions, and the atomic structural models are schematically shown in Figure 1.

The molecular dynamic (MD) simulations were carried out by the Large scale Atomic/Molecular Massively Parallel Simulator (LAMMPS) [20] codes from Sandia National Laboratories. The embedded atom method (EAM) potential given by Mendelev et al. [21] was employed. This EAM potential has been confirmed to successfully reproduce the physical properties of both perfect and defective crystals [11,22,23,24,25], as well as the deformations caused by twinning and dislocation slip in BCC-Fe nanowires [11,12,26]. All the MD simulations were conducted using the three-dimensional periodic boundary conditions. The energy minimization was performed by the conjugate gradient method to obtain the stable and/or equilibrium atomic structures. Before loading the tensile strain, the systems were relaxed for 300 ps in the NPT ensemble [7] to stabilize the system at the prescribed temperatures. The atomic positions were updated using the velocity Verlet algorithm [12] with a time step of 0.05 ps. After the equilibrium, the structural model was stretched and deformed at a constant strain rate along the z axis, while the x axis and the y axis maintained zero pressure. The simulation results were visualized using the OVITO [27] package, where the dislocation extraction algorithm (DXA) [28] technique was used to capture and trace the variations in dislocations and atomic structures.

## 3. Results

### 3.1. The Mechanical Responses of BCC-Fe

**Figure 2:**
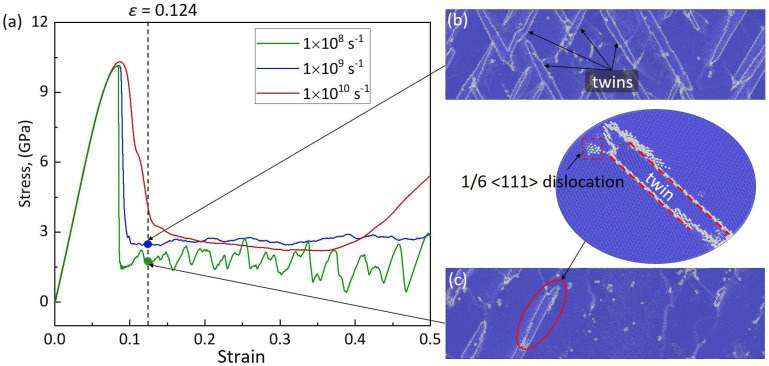
(**a**) The tensile stress–strain (*σ*-*ε*) curves of the <100>-BCC-Fe deformed at a given temperature of *T* = 300 K and different strain rates of $\dot{\varepsilon }$ = 1 × 10^8^ s^−1^, 1 × 10^9^ s^−1^, and 1 × 10^10^ s^−1^, respectively. The atomic configurations at a strain of *ε* = 0.124 with strain rates of (**b**) $\dot{\varepsilon }$ = 1 × 10^9^ s^−1^ and (**c**) 1 × 10^8^ s^−1^, respectively. Note that, in this and subsequent figures, the blue region represents the BCC structure and the white region represents other atomic structures.

Figure 2a shows the tensile stress–strain curves of the <100>-oriented BCC-Fe at different strain rates ($\dot{\varepsilon }$ = 1 × 10^8^ s^−1^, 1 × 10^9^ s^−1^, and 1 × 10^10^ s^−1^) and given temperature (*T* = 300 K) conditions. The stress reaches its peak value at the end of the elastic deformation stage, and then drops sharply due to yielding. During plastic deformation, the stress–strain curves present uniform oscillations in a near constant low stress level, which is a typical feature commonly observed in the tensile deformation of BCC-Fe [12,13,29]. The corresponding values of stress and strain at the yield point, along with the average flow stress (i.e., the mean stress of uniform oscillations after yielding) and the Young’s modulus, are listed in Table 1. This shows that, with the strain rate increasing from $\dot{\varepsilon }$ = 1 × 10^8^ s^−1^ to 1 × 10^10^ s^−1^, the strain at the yield point (*ε_y_* = 0.085 ± 0.0015) and the Young’s modulus (*E* = 145.76 ± 0.2 GPa) are almost unchanged, while the yield stress *σ_y_* and average flow stress *σ_f_* increase by 0.16 GPa and 0.82 GPa, respectively. The larger the strain rate, the shorter the time to respond to deformation, which makes the strain more localized and thus requires a larger stress to overcome the stress concentration.

**Figure 3:**
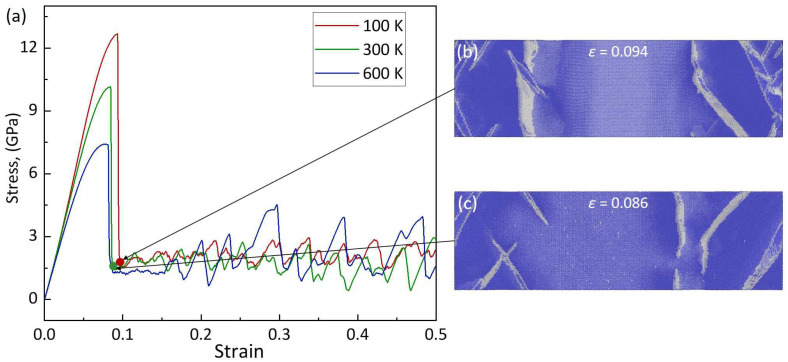
(**a**) The stress–strain (*σ*-*ε*) curves for the <100>-BCC-Fe deformed at a constant strain rate of $\dot{\varepsilon }$ = 1 × 10^8^ s^−1^, and the temperature of *T* = 100 K, 300 K, and 600 K, respectively. The atomic configurations under (**b**) *ε* = 0.094, *T* = 100 K and (**c**) *ε* = 0.086, *T* = 300 K conditions.

The tensile stress–strain curves for the <100>-oriented BCC-Fe at different temperatures (*T* = 100 K, 300 K, and 600 K) and a given strain rate ($\dot{\varepsilon }$ = 1 × 10^8^ s^−1^) are shown in Figure 3a. As listed in Table 1, with the temperature increasing from *T* = 100 K to 600 K, the yield stress decreases from *σ_y_* = 12.68 GPa to 7.43 GPa; the according strain decreases from *ε_y_* = 0.093 to 0.077, the average flow stress decreases from *σ_f_* = 2.41 GPa to 1.33 GPa, and the Young’s modulus decreases from *E* = 160.59 GPa to 132.19 GPa. In particular, in the elastic deformation regime, the stress of the <100>-oriented BCC-Fe increases linearly with the increasing strain at 100 K and 300 K, while the stress–strain curve shows slightly nonlinear behavior at 600 K. The reason for this is attributed to the serious thermal-induced atomic bonding vibrations, which lead to the easy deformation of the Fe-Fe bond, and thus a nonlinear stress–strain correlation, before yielding at high temperature conditions [6,11].

**Figure 4:**
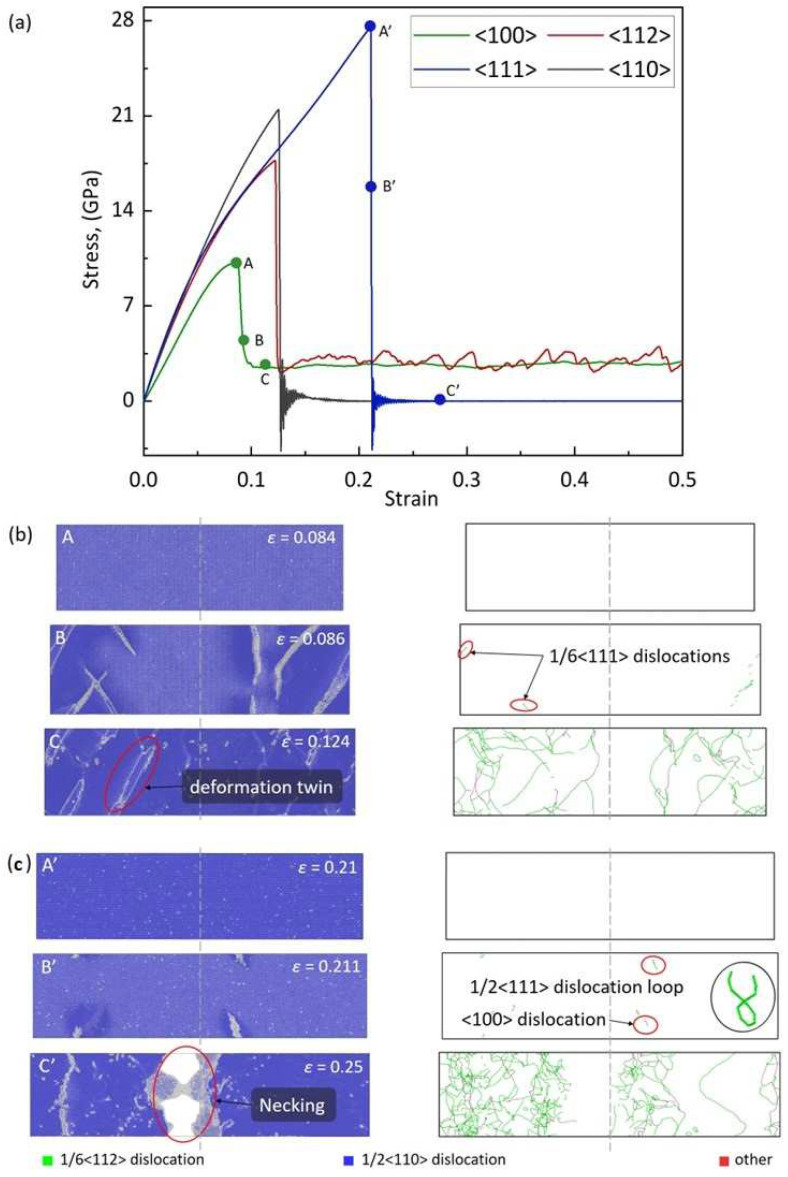
The tensile curves, atomic configurations, and dislocation distribution snapshot of BCC-Fe under $\dot{\varepsilon }$ = 1 × 10^8^ s^−1^ and *T* = 300 K conditions. (**a**) The tensile stress–strain (*σ*-*ε*) curves of BCC-Fe along <100>, <112>, <110>, and <111> orientations. The snapshot for atomic configurations and dislocation distribution of (**b**) the <100>-oriented BCC-Fe at different strain values (A: *ε* = 0.084, B: *ε* = 0.086 and C: *ε* = 0.124) and (**c**) the <111>-oriented BCC-Fe at different strain values (A’: *ε* = 0.21, B’: *ε* = 0.211 and C’: *ε* = 0.25), respectively.

Accordingly, we chose *T* = 300 K and $\dot{\varepsilon }$ = 1 × 10^8^ s^−1^ in subsequent MD simulations to investigate the mechanical behaviors along different crystal directions of BCC-Fe, aiming at exploring its orientation-dependent mechanical behaviors. Figure 4a shows the tensile stress–strain curves along <100>, <112>, <110>, and <111> orientations of BCC-Fe. As reflected, the mechanical responses exhibit obvious anisotropic characteristics, which is apparent in the elastic deformation stage. This confirms the large elastic anisotropic characteristics of BCC-Fe [12]. The <100>-oriented BCC-Fe presents the smallest elastic deformation regime, followed by increases in a sequence of <112>, <110>, and <111> orientations. During the elastic deformation, BCC-Fe exhibits linear behavior under small-strain conditions, and then nonlinear behavior under high-strain conditions. At the end of the elastic deformation stage, the stress drops and yielding occurs. The <100>- and <112>-oriented BCC-Fe have near constant stress in response to progressive plastic deformation. However, for the <111>- and <110>-oriented BCC-Fe, the stress drops abruptly to almost zero after yielding. As shown in Table 1, the <100>-oriented BCC-Fe has the minimum Young’s modulus of *E* = 147.56 GPa, followed by <112>, <110>, and <111> orientations with *E* = 219.45 GPa, 220.56 GPa, and 244.84 GPa, respectively. The <100>-oriented BCC-Fe has the lowest yield stress of *σ_y_* = 10.15 GPa, while the <111>-oriented BCC-Fe has the highest value of *σ_y_* = 27.57 GPa. The yield stress for other orientations varies between these two values, i.e., *σ_y_* = 17.58 GPa for the <112> orientation and *σ_y_* = 21.48 GPa for the <110> orientation. The according tensile strain at the yield point increases in the sequence of <100>-, <112>-, <110>-, and <111>-oriented BCC-Fe. These results are in good agreement with those available values predicted from ab initio calculations and other MD simulations reported in the literature [12,30,31], as shown in Table 1.

### 3.2. The Plastic Deformation Mechanisms of BCC-Fe

**Figure 5:**
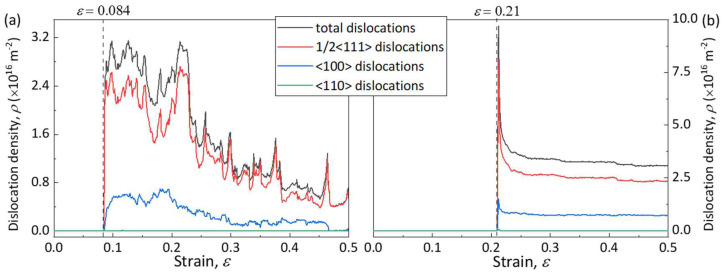
Variations in dislocation density as a function of strain for BCC-Fe at $\dot{\varepsilon }$ = 1 × 10^8^ s^−1^ and *T* = 300 K tensile conditions. (**a**) The <100>-oriented BCC-Fe and (**b**) the <111>-oriented BCC-Fe. The black, red, blue, and green curves represent the total dislocation density, along with the 1/2<111>, <110>, and <100> partial dislocations, respectively.

**Figure 6:**
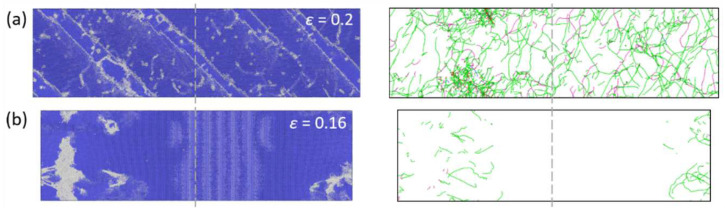
The atomic configurations and dislocation evolution snapshot of BCC-Fe under $\dot{\varepsilon }$ = 1 × 10^8^ s^−1^ and *T* = 300 K conditions. The snapshot for atomic configurations and dislocation distribution of (**a**) the <112>-oriented BCC-Fe at a strain of *ε* = 0.2 and (**b**) the <110>-oriented BCC-Fe at *ε* = 0.16.

Figure 4b shows a snapshot of the atomic configurations and dislocation evolution upon deformation of the <100>-oriented BCC-Fe at different magnitudes of strain under *T* = 300 K and $\dot{\varepsilon }$ = 1 × 10^8^ s^−1^ conditions. At the yield point where the strain is *ε_y_* = 0.084, there are partial BCC lattice types transiting into other types. When the strain increased to *ε* = 0.086, the nucleation of twins leads to a sharp drop in stress. Further stretching induces the occurrence of twinning expansion, twinning boundary migration, and annihilation, thus resulting in continuous deformation at a near constant stress level. The atomic configurations during plastic deformation are similar to that at a strain of *ε* = 0.124 (see Figure 2c), where the deformation twinning structures caused by the 1/6 <111> partial dislocations can be observed. After yielding, the repeated nucleation and slip of the 1/6 <111> partial dislocations lead to the growth of deformation twinning and an increase in dislocation density. The <100>-oriented BCC-Fe presents uniform oscillations at a constant stress level. The dislocation density variations with strain of the <100>-oriented BCC-Fe are shown Figure 5a, where a rapid increase in total dislocation density occurs after yielding. With tensile proceeding, the dislocations escape from the bulk to the surface and finally disappear into the system, resulting in a decrease in total dislocation density. Such a deformation twinning-dominated mode and mechanism are analogous to the <112>-oriented BCC-Fe, as can be seen in Figure 6a.

A similar analysis of the atomic structures indicates that the plastic deformation at different temperatures and strain rates of the <100>-oriented BCC-Fe is consistently dominated by the deformation twinning mode. Figure 2b,c show the atomic configurations at *ε* = 0.124 of the <100>-oriented BCC-Fe under $\dot{\varepsilon }$ = 1 × 10^9^ s^−1^, $\dot{\varepsilon }$ = 1 × 10^8^ s^−1^, and a given temperature of *T* = 300 K conditions. Compared with the tensile deformation at a strain rate of $\dot{\varepsilon }$ = 1 × 10^8^ s^−1^, the proportion of twinning defects in the crystal deformed under $\dot{\varepsilon }$ = 1 × 10^9^ s^−1^ conditions increases significantly, which leads to an increase in deformation resistance. Thus, a higher flow stress is required to overcome the increased deformation resistance, which results in a high yield strength and an increased flow stress. Figure 3b,c show the atomic configurations of the <100>-oriented BCC-Fe after yielding upon tensile deformation under *T* = 100 K, 300 K and $\dot{\varepsilon }$ = 1 × 10^8^ s^−1^ conditions. In turns out that the tensile deformation at a high *T* condition can benefit the initiation of deformation twinning in BCC-Fe. As exemplified, under *T* = 300 K, the deformation twinning defects begin to appear at a value of strain of *ε* = 0.086, which is earlier than that of *ε* = 0.094 under *T* = 100 K. Thus, the increase in temperature can promote deformation twinning formation in BCC-Fe. Accordingly, the increases in both the strain rate and temperature can provide impetus for the tensile deformation of the <100>-oriented BCC-Fe via twinning mode, but the plastic deformation mechanism is not affected.

Figure 4c shows the snapshot of the atomic configuration and dislocation distribution of the <111>-oriented BCC-Fe deformed under *T* = 300 K and $\dot{\varepsilon }$ = 1 × 10^8^ s^−1^ conditions. The yielding occurs at a value of strain of *ε* = 0.21, and other types of crystal lattice appear in the <111>-oriented BCC-Fe system. As reflected by B’ in Figure 4c, the dislocations start to nucleate at the corner sites and the 1/2<111> dislocation loops appear at a value of strain of *ε* = 0.211, which leads to a sharp drop in stress from 27.57 GPa to 0 GPa. Once the dislocation loops form without obstacles, they can easily extend and spread along different directions. With the increasing strain, a large number of straight screw dislocations accumulate, resulting in a sharp increase in dislocation density in the <111>-oriented BCC-Fe system (see Figure 5b). In general, the edge dislocations in BCC crystals have higher mobility than the screw dislocations [32]. With the tensile deformation proceedings, most edge dislocations escape from the bulk to the crystal surface, resulting in a decrease in dislocation density. Meanwhile, the activation of multiple slips leads to a neck formation in the <111>-oriented BCC-Fe, as reflected by C’ in Figure 4c. Accordingly, the tensile deformation of the <111>-oriented BCC-Fe is dominated by the dislocation slip, which is similar to that of the <110>-oriented BCC-Fe, as can be seen in Figure 6b.

## 4. Discussions

The above results and analysis show the apparent orientation-dependent mechanical behaviors of BCC-Fe. The variations in atomic configurations during the tensile deformation of BCC-Fe along different orientations are dominated by different plastic deformation mechanisms, which induce the anisotropy of mechanical properties. Then, how do the deformation mechanisms reflect the orientation-dependent mechanical properties of BCC-Fe? It has been reported in previous work [14] that the plastic deformation can be considered an atomic kinetic process driven by thermodynamics, and the mechanical responses can be described by the deformation thermo-kinetic synergy. In what follows, the underlying physics behind the anisotropic mechanical behaviors of BCC-Fe are discussed from the perspective of thermo-kinetic synergy for dislocation motion.

The evolution of defects such as dislocations and twins during plastic deformation governs the orientation-dependent mechanical behaviors of BCC-Fe [14,17]. The nucleation and growth of the deformation twinning are trigged by the 1/6<111> partial dislocations [33,34,35]. Thus, the orientation-dependent mechanical behaviors of BCC-Fe are derived from the nucleation and slip of different type dislocations, which determines the strength–ductility trade-off correlation. Specifically, the yield strength of BCC-Fe is related to the nucleation mechanism of dislocations. In general, the energy required for the nucleation of the partial dislocations is lower than that of full dislocations [32,36,37]; thus, the energy barrier for the twin deformation induced by the partial dislocations that must be overcome is lower than that for the full dislocation slip. Accordingly, the plastic deformation of the <100>- and <112>-oriented BCC-Fe, which is dominated by deformation twinning, requires that the smaller resistance after yielding be overcome, thus corresponding to a lower yield strength. In contrast, the plastic deformation of the <110>- and <111>-oriented BCC-Fe is governed by the slip of half <111> full dislocation. Thus, the <110>- and <111>-oriented BCC-Fe requires a larger driving force to overcome the deformation resistance, and this corresponds to a higher yield strength.

When the dislocation nucleation is driven by thermal activation [38], the thermodynamic driving force (Δ*G*) is defined as the difference between applied stress (Δ*G_σ_*) and slip resistance (Δ*G_τ_*) [39,40]:

(1) $$∆G=∆G_{\sigma }-∆G_{\tau }=\sigma_{f}(1-C/M\alpha )$$

where Δ*G_σ_* = *σ_f_* is the flow stress given by *σ_f_* = *MαGbρ_t_*^1/2^ [41]; Δ*G_τ_* = *CGbρ_t_*^1/2^, with *b* being the Burgers vector, *G* the shear modulus, *M* the Taylor factor, and *α* and *C* the geometrical factors depending on the type and distribution of the interacting dislocations (e.g., *b* = 2.48 Å*, G* = 65 GPa, *M* = 2.8, *α* = 0.38, and *C* = 0.393 for BCC-Fe) [15,16,17,40]. Based on the results analyzed in the above section, the driving force at the yield point of the <100>-oriented BCC-Fe is Δ*G* = 6.395 GPa and the yield strength *σ_y_* = 10.15 GPa, while those of the <111>-oriented BCC-Fe are Δ*G* = 17.37 GPa and *σ_y_* = 27.57 GPa.

Following the Orowan equation of $\dot{\varepsilon }$ = *bv_d_ρ_m_*/*M* [41], the strain *ε* is associated with the dislocation velocity *v_d_* and the mobile dislocation density *ρ_m_*. The dislocation velocity is given by *v_d_* = *v*_0_*λ*exp[-*Q*/(*kT*)] [40], where *Q* is the effective activation energy for dislocation glide, *λ* the mean spacing of dislocation, *v*_0_ the attack frequency, and *k* the Boltzmann constant. In combination with the Orowan equation [41] and dislocation velocity equation, the correlation between the plastic strain (*ε*) and activation energy of the dislocation slip (*Q*) is deduced as follows:

(2) $$Q=kTln\left(\frac{v_{0}\varepsilon M}{v_{d}\rho_{m}b}\right)$$

Accordingly, at the value of strain of *ε*_<100>_ = 0.5 and *ε*_<111>_ = 0.21 for the <100>- and <111>-oriented BCC-Fe, the effective activation energy is calculated as *Q* = 11.95 KJ/mol and 6.45 KJ/mol, respectively (where *v*_0_ = 1.36 × 10^13^/s and *v_d_* = 2.4 × 10^5^/s for BCC-Fe) [40]. Thus, for the <100>-oriented BCC-Fe, the small driving force Δ*G* corresponds to the low yield strength *σ_y_*, while the high activation energy *Q* corresponds to a large plastic strain *ε*. Meanwhile, for the <111>-oriented BCC-Fe, the large Δ*G* corresponds to the high *σ_y_*, while the low *Q* corresponds to the small *ε*. As such, the orientation-dependent mechanical behaviors of BCC-Fe can be understood from the thermo-kinetic synergy for dislocation evolution during plastic deformation.

Interestingly, the thermodynamic driving force (Δ*G*) and effective activation energy (*Q*) present a trade-off correlation similar to the yield strength (*σ_y_*) and plasticity (*ε*) of BCC-Fe. For example, the <111>-oriented BCC-Fe exhibits a high yield strength, signifying that a high flow stress is required to driven the deformation, and this corresponds to the enhancement of driving force Δ*G* (see Equation (1)). According to *Q* = *Q*_0_[1 − (*σ_f_*/*σ*)*^p^*]*^q^* [40] (where 0.5 ≤ *p* ≤ 1.0 and 1.0 ≤ *q* ≤ 2.0 are the exponents, *σ* is the threshold resistance, and *Q*_0_ is the total energy needed to overcome short-range obstacles without applied shear stress, 0.05 ≤ *Q*_0_/(G*b*^3^) ≤ 2.0), the effective activation energy *Q* for dislocation slip is inversely proportional to the flow stress. The high flow stress is accompanied with a low *Q*, which corresponds to a low ductility (see Equation (2)). Accordingly, the thermo-kinetic synergy of dislocation motion is responsible for the strength–ductility trade-off correlation of BCC-Fe.

## 5. Conclusions

In conclusion, the orientation-dependent mechanical behaviors of BCC-Fe, and the reasons behind them, were investigated in light of the thermo-kinetic synergy for dislocation motion. The mechanical responses and deformation mechanisms of BCC-Fe along different crystal orientations were analyzed on the basis of the atomic configurations and tensile curves obtained from MD simulations. The results indicate that the anisotropic mechanical behaviors and the strength–ductility exclusive correlation are derived from the synergistic effects between thermodynamics and kinetics of dislocation motion. The plastic deformation of the <100>- and <112>-oriented BCC-Fe is dominated by deformation twinning, while that along the <110> and <111> orientation is governed by the dislocation slip. In combination with Equations (1) and (2), the strength and ductility of BCC-Fe depend on the thermodynamic driving force and kinetic energy barrier for dislocation motion. The small driving force (Δ*G* = 6.395 GPa) and high energy barrier (*Q* = 11.95 KJ/mol) of the <100>-oriented BCC-Fe correspond to the low yield strength (*σ_y_* = 10.15 GPa) and large ductility (*ε* = 0.5), while the large Δ*G* (17.37 GPa) and low *Q* (6.45 KJ/mol) of the <111>-oriented BCC-Fe correspond to the high *σ_y_* (27.57 GPa) and small *ε* (0.21). Thus, high strength is associated with a large Δ*G* (for <111>-oriented BCC-Fe), and large ductility is related to high *Q* (for <100>-oriented BCC-Fe), and vice versa. The obtained results confirmed the expectations and assumptions of our previous works [4,39,40,41]. The present work offers a new viewpoint on thermo-kinetic synergy upon deformation in order to comprehend the orientation-dependent mechanical behaviors and the strength–ductility trade-off phenomenon of BCC-Fe and its alloys. The MD simulations adopted in the present work can also be applied to the study of deformation behaviors of other metallic alloys, and the underlying physical origin of the strength and ductility paradox of metallic alloys can be explored by combining Equations (1) and (2), thus providing a new method for the design of advanced metallic structural materials.

## Figures and Tables

**Table 1:** The mechanical properties of BCC-Fe under different temperatures *T* (K), strain rates $\dot{\varepsilon }$ (s^−1^), and crystallographic orientations <*uvw*>, including the yield stress *σ_y_* (GPa), the strain at the yield point *ε_y_*, the average flow stress *σ_f_* (GPa), and the Young’s modulus *E* (GPa), along with those values referenced from the literature.

	*T* (K)		$\dot{\varepsilon }$ (s^−1^)		<*uvw*>				Reference
	100	600	10^9^	10^10^	<100>	<110>	<111>	<112>	
*σ_y_*	12.68	7.43	10.16	10.31	10.15	21.48	27.57	17.58	This work
					12.3	22.7	27.2	17.1	[12]
					12.6	-	27.3	-	[30]
*ε_y_*	0.093	0.0765	0.085	0.0865	0.084	0.125	0.21	0.122	This work
					0.08	0.146	0.245	0.134	[12]
*σ_f_*	2.41	1.33	2.87	2.43	1.61	-	-	2.94	This work
*E*	160.59	132.19	145.81	145.76	147.56	220.56	244.84	219.45	This work
					164	195	-	212	[12]
					155	-	285	-	[30]
					-	210	-	227	[31]

## Data Availability

Data are contained within the article.

## References

[1] Dehm G., Jaya B.N., Raghavan R., Kirchlechner C. (2018). Overview on micro- and nanomechanical testing: New insights in interface plasticity and fracture at small length scales. Acta Mater..

[2] Souq S.M.N., Ghasemi F.A., Fakhrabadi M.M.S. (2023). Effects of Various Cross Sections on Elastoplastic Behavior of Fe Nanowires under Tension/Compression. J. Mater. Eng. Perform..

[3] Sedona F., Di Marino M., Forrer D., Vittadini A., Casarin M., Cossaro A., Floreano L., Verdini A., Sambi M. (2012). Tuning the catalytic activity of Ag(110)-supported Fe phthalocyanine in the oxygen reduction reaction. Nat. Mater..

[4] Du J., Liu Y., Zhang Z., Xu C., Gao K., Dai J., Liu F. (2023). Mechanical behaviors of metallic alloys dominated by thermo-kinetic synergistic effects upon materials processing. Mater. Today Commun..

[5] Du J., Liu Y., Zhang Z., Shang S.-L., Li H., Liu Z.-K., Liu F. (2023). Deformation behaviors in light of dislocation core characteristics with respect to the compositional-dependent misfit potentials of aluminum alloys. J. Mater. Res. Technol..

[6] Zhu Y., Liao X. (2004). Retaining ductility. Nat. Mater..

[7] Husain A., La P., Hongzheng Y., Jie S. (2020). Molecular Dynamics as a Means to Investigate Grain Size and Strain Rate Effect on Plastic Deformation of 316 L Nanocrystalline Stainless-Steel. Materials.

[8] Liu Y., Du J., Shang S., Zhang A., Xiong S., Liu Z.-K., Liu F. (2023). Insights into plastic deformation mechanisms of austenitic steels by coupling generalized stacking fault energy and semi-discrete variational Peierls-Nabarro model. Prog. Nat. Sci. Mater..

[9] Harding J. (1967). The yield and fracture behaviour of high-purity iron single crystals at high rates of strain. Proc. Phys. Soc. Lond. Ser. A.

[10] Wang J., Zeng Z., Weinberger C.R., Zhang Z., Zhu T., Mao S.X. (2015). In situ atomic-scale observation of twinning-dominated deformation in nanoscale body-centred cubic tungsten. Nat. Mater..

[11] Healy C.J., Ackland G.J. (2014). Molecular dynamics simulations of compression-tension asymmetry in plasticity of Fe nanopillars. Acta Mater..

[12] Sainath G., Choudhary B.K. (2016). Orientation dependent deformation behaviour of BCC iron nanowires. Comput. Mater. Sci..

[13] Sainath G., Choudhary B.K., Jayakumar T. (2015). Molecular dynamics simulation studies on the size dependent tensile deformation and fracture behaviour of body centred cubic iron nanowires. Comput. Mater. Sci..

[14] Huang L., Lin W., Zhang Y., Feng D., Li Y., Chen X., Niu K., Liu F. (2020). Generalized stability criterion for exploiting optimized mechanical properties by a general correlation between phase transformations and plastic deformations. Acta Mater..

[15] Du J., Zhang Z., Liu Y., Shao Q., Zhang A., Xiong S., Liu F. (2023). Strength-ductility trade-off modulated by thermo-kinetic synergy of heat-treatable aluminum alloys. J. Mater. Res. Technol..

[16] He Y., Song S., Du J., Peng H., Ding Z., Hou H., Huang L., Liu Y., Liu F. (2022). Thermo-kinetic connectivity by integrating thermo-kinetic correlation and generalized stability. J. Mater. Sci. Technol..

[17] Ding Z., Hou H., Liu W., Kan J., Sun Y., Liu F. (2023). Quantitative determination of the generalized stability of Fe-based binary alloys. Materialia.

[18] Liu F. (2023). Nucleation/growth design by thermo-kinetic partition. J. Mater. Sci. Technol..

[19] Davey W.P. (1925). Precision Measurements of the Lattice Constants of Twelve Common Metals. Phys. Rev..

[20] Plimpton S. (1995). Fast parallel algorithms for short-range molecular dynamics. J. Comput. Phys..

[21] Mendelev M.I., Han S., Srolovitz D.J., Ackland G.J., Sun D.Y., Asta M. (2003). Development of new interatomic potentials appropriate for crystalline and liquid iron. Philos. Mag..

[22] Domain C., Monnet G. (2005). Simulation of Screw Dislocation Motion in Iron by Molecular Dynamics Simulations. Phys. Rev. Lett..

[23] Abe Y. (2013). Application of hyper-molecular dynamics to self-interstitial diffusion in α-iron. Comput. Mater. Sci..

[24] Gordon P.A., Neeraj T., Li Y., Li J. (2010). Screw dislocation mobility in BCC metals: The role of the compact core on double-kink nucleation. Model. Simul. Mater. Sci. Eng..

[25] Queyreau S., Marian J., Gilbert M.R., Wirth B.D. (2011). Edge dislocation mobilities in bcc Fe obtained by molecular dynamics. Phys. Rev. B.

[26] Dutta A. (2017). Compressive deformation of Fe nanopillar at high strain rate: Modalities of dislocation dynamics. Acta Mater..

[27] Stukowski A. (2010). Visualization and analysis of atomistic simulation data with OVITO—The Open Visualization Tool. Model. Simul. Mater. Sci. Eng..

[28] Stukowski A., Bulatov V.V., Arsenlis A. (2012). Automated identification and indexing of dislocations in crystal interfaces. Model. Simul. Mater. Sci. Eng..

[29] Wu Q., Wang Y., Han T., Wang H., Han L., Bao L. (2021). Molecular Dynamics Simulations of the Effect of Temperature and Strain Rate on the Plastic Deformation of Body-Centered Cubic Iron Nanowires. J. Eng. Mater. Technol..

[30] Friák M., Šob‖ M., Vitek V. (2003). *Ab initio* calculation of tensile strength in iron. Philos. Mag..

[31] Dieter G.E. (1976). Mechanical Metallurgy.

[32] Kaufmann D., Mönig R., Volkert C.A., Kraft O. (2011). Size dependent mechanical behaviour of tantalum. Int. J. Plast..

[33] Saha S., Abdul Motalab M., Mahboob M. (2017). Investigation on mechanical properties of polycrystalline W nanowire. Comput. Mater. Sci..

[34] Li L., Han M. (2017). Molecular dynamics simulations on tensile behaviors of single-crystal bcc Fe nanowire: Effects of strain rates and thermal environment. Appl. Phys. A.

[35] Park H.S., Gall K., Zimmerman J.A. (2006). Deformation of FCC nanowires by twinning and slip. J. Mech. Phys. Solids.

[36] Li S., Ding X., Deng J., Lookman T., Li J., Ren X., Sun J., Saxena A. (2010). Superelasticity in bcc nanowires by a reversible twinning mechanism. Phys. Rev. B.

[37] Duesbery M.S., Vitek V. (1998). Plastic anisotropy in bcc transition metals. Acta Mater..

[38] Galindo-Nava E.I., Rae C.M.F. (2016). Microstructure-sensitive modelling of dislocation creep in polycrystalline FCC alloys: Orowan theory revisited. Mater. Sci. Eng. A.

[39] Rollett A.D., Kocks U.F. (1993). A Review of the Stages of Work Hardening. Solid Stat. Phenom..

[40] Argon A. (2008). Strengthening Mechanisms in Crystal Plasticity.

[41] Orowan E. (1940). Problems of plastic gliding. Proc. Phys. Soc..

